# Essays and Addresses

**Published:** 1853-09

**Authors:** 


### ESSAYS AND ADDRESSES.

A number of excellent essays and addresses have accumulated on our table, of which we would be glad to speak at length if we had the leisure and room. As it is, we can only notice them very briefly.

"*Essays on Asylums for persons of unsound mind,*" is the title of a neat pamphlet, by Dr. John M. Galt, of Williamsburg, Va., which has passed to a second edition. Dr. Galt feels a noble enthusiasm in the cause of the insane which, for many years, has engrossed his thoughts and time, and in these essays he submits the results of his experience and inquiries in regard to the retreats for this unfortunate class of our species. Their wide diffusion would be a public benefit.

"*Respiration subservient to Nutrition.*" A thesis, by E. Leigh, M. D., of Townsend, Mass., bears this title. It is well written, and the argument is ingenious. The purpose of respiration, he attempts to show, is not so much to maintain animal heat as to complete nutrition. There is no doubt the objects of respiration are manifold, and that one of them relates to the last named function. It is a matter of common observation, that animals with large chests are those most remarkable for the activity of the nutritive process.

"*An Essay on the unity of Disease,*" read by Dr. H. Backus, before the Alabama State Medical Association, evinces thought and reading and is calculated to promote inquiry.

Dr. J. B. S. Jackson, of Boston, has favored us with a copy of his admirable address on *Morbid Anatomy*, delivered before the Massachusetts Medical Society. The author shows himself at every step master of his subject, and the reader who does not lay down the address with a feeling that he has been instructed by its perusal, must be an adept in such studies.

Dr. John W. Richardson, of Rutherford, Ten., pronounced an eloquent address to the graduates of the University of Nashville, which has been published. Few men are better qualified than Dr. R. to treat of the "difficulties and responsibilities of the Physician," which is the subject of his interesting address.

"*Retrospective, Perspective, and Prospective View af Medicine.*" An address delivered before the Tennessee State Medical Society, by Dr. John M. Watson, of Nashville, bears the above title. Dr. W. reviews the progress of medicine, examines its present condition, and shows that it has made great advances. His address contains some fair hits at quackery and many just sentiments.

